# Comparison of Four Control Methods for a Five-Choice Assistive Technology

**DOI:** 10.3389/fnhum.2018.00228

**Published:** 2018-06-06

**Authors:** Sebastian Halder, Kouji Takano, Kenji Kansaku

**Affiliations:** ^1^Systems Neuroscience Section, Department of Rehabilitation for Brain Functions, Research Institute of National Rehabilitation Center for Persons with Disabilities, Tokorozawa, Saitama, Japan; ^2^Department of Molecular Medicine, University of Oslo, Oslo, Norway; ^3^Brain Science Inspired Life Support Research Center, The University of Electro-Communications, Tokyo, Japan; ^4^Department of Physiology and Biological Information, Dokkyo Medical University School of Medicine, Tochigi, Japan

**Keywords:** BCI, EEG/ERP, assistive technology, eye-tracking, visual stimulation, auditory stimulation, tactile stimulation

## Abstract

Severe motor impairments can affect the ability to communicate. The ability to see has a decisive influence on the augmentative and alternative communication (AAC) systems available to the user. To better understand the initial impressions users have of AAC systems we asked naïve healthy participants to compare two visual (a visual P300 brain-computer interface (BCI) and an eye-tracker) and two non-visual systems (an auditory and a tactile P300 BCI). Eleven healthy participants performed 20 selections in a five choice task with each system. The visual P300 BCI used face stimuli, the auditory P300 BCI used Japanese Hiragana syllables and the tactile P300 BCI used a stimulator on the small left finger, middle left finger, right thumb, middle right finger and small right finger. The eye-tracker required a dwell time of 3 s on the target for selection. We calculated accuracies and information-transfer rates (ITRs) for each control method using the selection time that yielded the highest ITR and an accuracy above 70% for each system. Accuracies of 88% were achieved with the visual P300 BCI (4.8 s selection time, 20.9 bits/min), of 70% with the auditory BCI (19.9 s, 3.3 bits/min), of 71% with the tactile BCI (18 s, 3.4 bits/min) and of 100% with the eye-tracker (5.1 s, 28.2 bits/min). Performance between eye-tracker and visual BCI correlated strongly, correlation between tactile and auditory BCI performance was lower. Our data showed no advantage for either non-visual system in terms of ITR but a lower correlation of performance which suggests that choosing the system which suits a particular user is of higher importance for non-visual systems than visual systems.

## 1. Introduction

Injuries or neurodegenerative diseases may lead to an interruption of the output of the central nervous system to the muscles. Ultimately, diseases such as amyotrophic lateral sclerosis (ALS) or injuries caused e.g., by brain-stem stroke may lead to the locked-in state (LIS), in which the affected person is conscious but will no longer be able to communicate without assistance (Plum and Posner, 1972; Storm et al., 2017; Juel et al., 2018). The causes of LIS may be diverse, nonetheless the requirement for a communication method is a common factor (Pels et al., 2017). A variety of augmentative and alternative communication (AAC) strategies that can restore communication are available ranging from eye-trackers to speech synthesis (Beukelman et al., 2013). Additionally, decoding biosignals for communication and control is possible using electrophysiological signals such as electromyogram (EMG), or, electroencephalogram (EEG) recordings for brain-computer interface (BCI) based control (Lesenfants et al., 2016; Käthner et al., 2017; Kawase et al., 2017). Also, to provide access to BCI technology for persons who lost vision, systems that are independent of muscle control and vision are needed. Such a system can be implemented by using event-related potentials (ERPs) elicited with non-visual stimulation to control a BCI (Furdea et al., 2009; Brouwer and van Erp, 2010).

As a number of different systems are available for persons with differing degrees of motor impairment, in particular systems that depend on vision and systems that do not, we wanted to compare the initial impression naïve users have of such systems. We chose two vision dependent systems, an eye-tracker and a visual P300 BCI, and two vison independent systems, an auditory and a tactile P300 BCI. There are other possibilities, such as motor imagery controlled BCIs (Müller-Putz et al., 2005) but comparing more systems is not feasible in one session and thus we chose four systems that could be used for the same five-choice selection task (eliminating or at least reducing the influence of the task on the users' impression).

Both the eye-tracker and the P300 BCI allow the user to make selections of symbols presented on a computer screen. Eye-trackers determine the point of gaze using cameras. The technology for this is available at a lower cost than EEG amplifiers, can be considered robust and have high acceptance rates among users (Ware and Mikaelian, 1987; Spataro et al., 2014). Selections are made by dwelling a the target element for a particular amount of time. Visual P300 BCIs rely on the fact that particular ERPs are elicited when the user differentiates between a rare and a frequent stimulus. This effect can be utilized to determine the element the user wants to selecting by presenting all stimuli in random pattern. Whenever the rare event (in case of the classic visual P300 BCI, the flash) occurs a P300 is elicited, when the frequent event (a non-attended element flashes) occurs the P300 ERP component is not elicited (Farwell and Donchin, 1988). Both visual P300 BCIs and eye-tracking systems have been successfully evaluated with persons with ALS and other forms of motor impairment (Nijboer et al., 2008; Spataro et al., 2014; Okahara et al., 2017; Utsumi et al., 2018). The degree of robustness both systems reach enable them to be used for complex tasks such as communication, accessing the internet and social networks or smart home control (Ball et al., 2010; Käthner et al., 2017). A previous comparison between eye-trackers and BCIs used in a web browsing task have shown the eye-tracker to have a lower workload than the BCI (Pasqualotto et al., 2015). A single case study by Käthner et al. (2015) reached similar conclusions when comparing an eye-tracker to an auditory BCI and an electrooculogram (EOG) selection task. More recently, a study comparing steady-state visually evoked potential (SSVEP) BCIs with an eye-tracker found higher accuracies of the BCI system for a high number of possible selections on the display at the same time (Suefusa and Tanaka, 2017). It is worth noting, that the participant in the study of Käthner et al. (2015), as well as participants in other studies (Fried-Oken et al., 2006; Spataro et al., 2014), stated that they used the eye-tracker based AAC method less frequently than face-to-face communication with a caregiver (indicating a preference for face-to-face communication). Face-to-face communication, eye-tracking and visual P300 BCIs all rely on the user being able to generate an overt control signal, such as moving the eyes, or attending to different visual stimuli. Depending on the injury or condition of the user, the ability to see may be lost. Thus, there is a requirement to develop methods that can make BCI communication accessible without relying on vision.

One method that is being developed to provide such a communication method are non-visual P300 BCIs. The most commonly used modalities are auditory and tactile stimulation (Furdea et al., 2009; Brouwer and van Erp, 2010; Onishi et al., 2017). The principle is the same as for the visual P300 BCIs: the user focuses on stimuli which occur rarely compared to the other stimuli and thus elicits a P300. The task becomes more difficult because the user cannot ignore the other stimuli as easily but has to do so by focussing attention without the ability to attenuate the other stimuli. Additionally, visually evoked potentials (VEPs) contribute to the classification performance in the case of visual BCIs. Overall, both these effects lead to a lower communication speed than with visual P300 BCIs and eye-trackers. In a comparison between visual, tactile and auditory stimulation in a LIS patient, Kaufmann et al. (2013a) showed that above chance classification was only possible using tactile stimulation. Later, Silvoni et al. (2016) showed the presence of tactile ERPs in a group of 14 ALS patients. Unfortunately, this is not alway true as in a single case study by Murguialday et al. (2011) with a non-responsive patient in the complete locked-in state (CLIS) only auditory but no tactile ERPs could be evoked. In a sample of five persons with motor impairments Halder et al. (2016a) were able to show that three of these five persons can learn to communicate with an auditory P300 BCI. To our knowledge, this has been shown only with healthy controls using tactile ERPs (van der Waal et al., 2012).

A reliable AAC method can have a profound impact on the the quality of life of a person with motor impairments. Thus, when presenting one of the available methods to a person it is important to ensure that this person can quickly gain control over the method and experiences as little frustration as possible. To make an informed choice on which method to present it is important to investigate how they compare to one another. As discussed in the previous paragraphs, the advantages and disadvantages of the different methods are not evident from the current literature. So far, we assume two things to be true. One, with intact vision systems using visual attention shifts will provide higher accuracy and speed than non-visual systems. And two, non-visual systems are needed for persons that lose the ability to see. Which non-visual system a user with intact vision and which non-visual system a user may prefer has not been thoroughly investigated. To better understand possible preferences we decided to investigate the initial impression users that are naïve to AAC systems and BCIs have when confronted with an eye-tracker, a visual, an auditory and a tactile P300 BCI the first time. We chose an interface that can be used to make one out of five selections with all four control methods. Five choices can easily be expanded to enable e.g., from 25 letters for spelling with the Latin alphabet as shown in Furdea et al. (2009). Thus, there is a practical application. Due to the number of systems we evaluated and the limitation of time only using five choices enabled us to ask the users to perform each possible selection several times.

## 2. Methods

Eleven healthy participants were asked to perform the same one out of five selection task with four different AAC systems.

### 2.1. Participants

Eleven healthy controls were recruited (six female, mean age 32.2 years). The study was approved by the institutional ethics committee at the National Rehabilitation Center for Persons with Disabilities and all participants provided written informed consent according to institutional guidelines. All experiments were carried out in accordance with the approved guidelines.

### 2.2. Procedure

**Figure 1:**
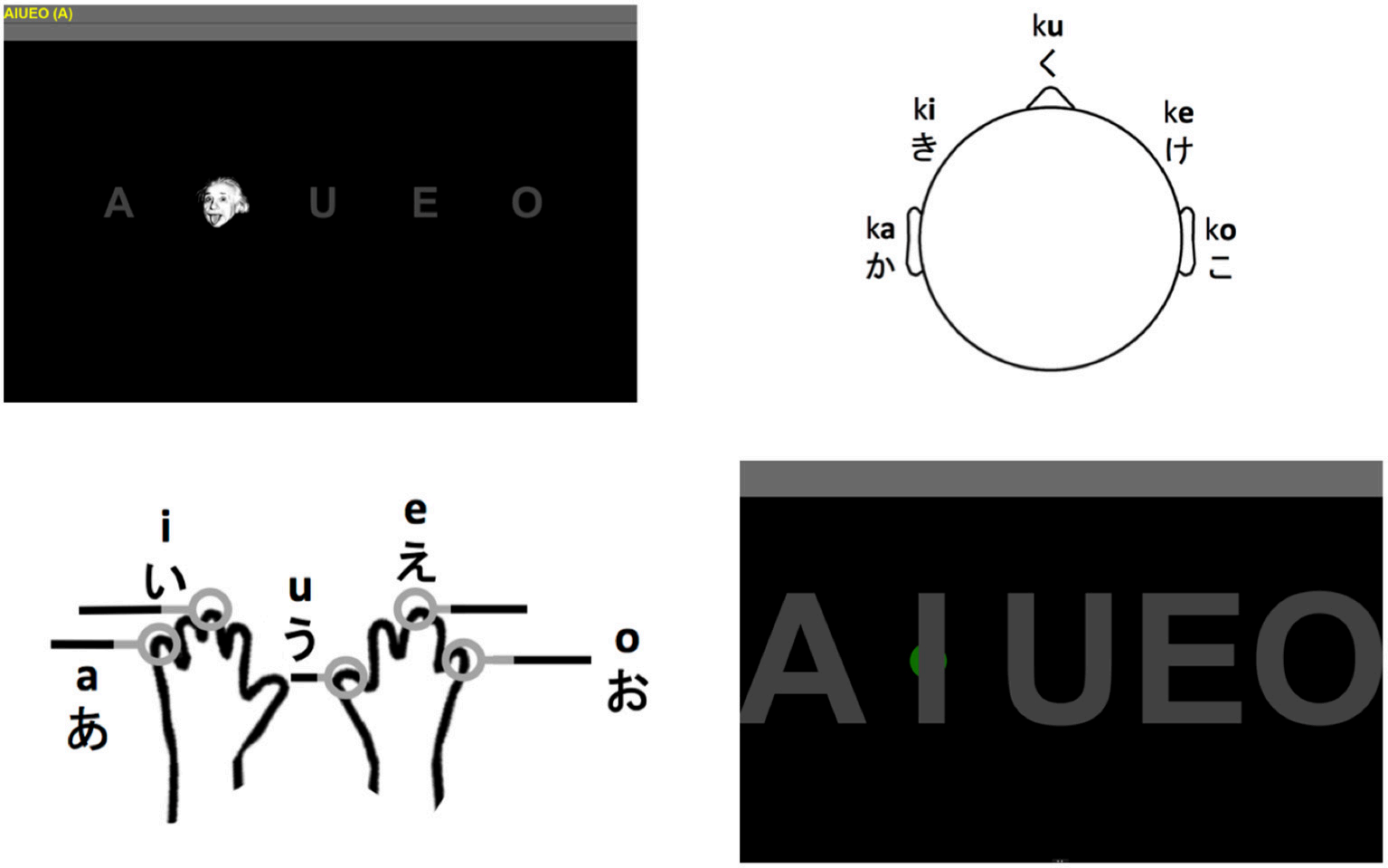
Visual P300 BCI interface using Einstein faces as stimuli (**top left**). The interface of the auditory P300 BCI using five stimuli originating from different directions (**top right**). The stimuli are the five Hiragana syllables ka, ki, ku, ke, and ko. The locations tactile stimulators were attached to and the corresponding vowels that could be selected with the tactile P300 BCI (**bottom left**; the hand outline image is a public domain work). The interface used for selection with the eye tracker (**bottom right**). The green dot visible behind the “I” showed the user the current gaze position. Selection was based on dwell time (minimum 3 s).

The interfaces used by the participants are shown in Figure 1 and could be used to select one of five possible targets (the vowels a, i, u, e, o). Participants were asked to select the five possible choices four times (total 20 selections). Thus all input modalities were configured to be used for identical tasks. Five choices have been shown to be feasible for auditory and tactile modalities. For example, five classes are sufficient to select either rows and columns from the matrix used the auditory speller experiments with the Latin alphabet or the vowels in the Hiragana syllabary (Furdea et al., 2009; Halder et al., 2016b). In the tactile domain, wheelchair control was shown to be possible with four classes (Kaufmann et al., 2014). Consequently, the same procedure can be used for communicating or mobility using a visual P300 BCI or an eye-tracker. All participants performed the tasks in the sequence visual, auditory, tactile P300 BCI and then eye-tracker (the interfaces are shown in Figure 1). The reasoning behind this was that the visual P300 BCI would take the shortest amount of time and be the easiest to understand. Additionally, over 90% of healthy people are able to control this type of BCI (Guger et al., 2009). Thus, if the participant is unable to control the visual P300 BCI it is a good indicator of misunderstanding the instructions. Considering that eye-trackers were shown to have a much lower workload and the limited group size, we concluded it would be acceptable always perform the eye-tracking task last as opposed to five before the BCI tasks and five after.

As indicated in Figure 1 (top left) the users were shown the five vowels of the current run on the top left of the screen and the current target in parenthesis behind those five vowels. This was identical for all four tasks.

#### 2.2.1. Visual AACs (visual P300 BCI and eye-tracker)

During the visual P300 BCI tasks the five gray letters on a black background were “flashed” by overlaying them with a human face (Figure 1, top left) which was shown to improve performance (Kaufmann et al., 2011). The participants were asked to focus on the target letter only and count the number of appearances of the face. The faces appeared for 62.5 ms with intervals of 125 ms. Each of the five letter was overlaid with the face 10 times (this is the number of stimulus presentations per target). Between letters the system paused for 3 s. In total, a selection with this system required between 3.9 s (one stimulus presentation per target) and 12.4 s (10 stimulus presentations per target).

The eye-tracker (Figure 1, bottom right) was first calibrated using the software provided with the Tobii EyeX eye-tracking system. Feedback during the eye-tracker task was provided in the form of a small gray circle which would follow gaze along the x-axis of the screen and remain fixed on the y-axis of the screen. The circle turned green if the user remained fixated in the area of the rectangle around the target. The target was then selected after 3 s which was indicated by an affirmative sound upon which the user was instructed to move to next target. If the system did not detect the user or the circle moved around continuously no selection was made until the user was detected or fixated a particular point again. Thus, the eye-tracker was the only system configured to use an asynchronous approach. The reasoning behind this was that it is very easy to implement based on the detection of the eyes and not using it would constitute and unfair advantage of the BCI systems.

#### 2.2.2. Non-visual AACs (auditory and tactile P300 BCI)

The auditory P300 BCI (Figure 1, top right) was based on the design presented in Halder et al. (2016b) but used only the five choice selecting task for the vowels. Thus, the participants put on headphones and heard the Japanese Hiragana syllables *ka, ki, ku, ke*, and *ko* in a random sequence. Stimuli were presented using Etymotic ER4 MicroPro (Etymotic Research, Inc., USA) earphones. The participants were asked to adjust the volume to their preference. As in Halder et al. (2016b), the five syllables were presented from five different virtual directions (see Figure 1, top right) by adapting the interaural time difference (ITD) and interaural level difference (ILD). The participants were asked to focus on the target direction and sound and count the number of appearances (which was set to 10). The time from the beginning of one stimulus to the beginning of the next was 375 ms (stimulus durations varied slightly, but the onset asynchrony was constant). The interval between selections was set to 3 s. In total, a selection with the auditory P300 BCI required between 4.9 s (one stimulus presentation per target) and 21.8 s (10 stimulus presentations per target).

In the tactile P300 BCI (Figure 1, bottom left) task the vowels corresponded to stimulators attached to the left little (a), left middle (i), right thumb (u), right middle (e) and right little finger (o) of the participants (see Figure 1, bottom left). These were again activated in a random sequence and the participant was asked to count the vibrations on the finger corresponding to the target. Each stimulator was activated 10 times with a duration of 250 ms and an interval of 250 ms. Shorter intervals made discriminating the stimuli difficult, therefore we chose to use a different setting than with the auditory BCI. The tactile stimulator was custom made and it was not possible to load more than the stimulation sequence for one letter sequence. Thus, the system was paused for loading another stimulus sequence between selections and this time could vary. Since this is a technical detail not inherent to tactile P300 BCIs we chose to use the same 3 s as pause between selections as with the other systems for purposes of calculating the information transfer rate (ITR). Then, the total selection time with the tactile BCI varied from 5.5 s (one repetition) to 28 s (10 repetitions).

All measurements were performed at the National Rehabilitation Center for Persons with Disabilities. Participants were seated approximately 1m away from a 24 inch computer screen in a shielded room.

The first 10 selections of the visual and auditory P300 BCI measurements were used to calibrate an stepwise linear discriminant analysis (SWLDA) classifier for online feedback. The participants could see their selections on the computer screen on the line below the target letters. Online feedback for the tactile BCI could not be provided at the time for technical reasons. The eye-tracker was calibrated before the measurement and feedback was provided for all selections.

### 2.3. Questionnaire

After completing the tasks the participants were asked the following questions about their experience.

1. How difficult/easy was it for you to control the eye tracker/visual/auditory/tactile P300 BCI on a scale from 0 to 10 (0 = very difficult, 10 = very easy)?
2. How tiring was it for you to control the eye tracker/visual/auditory/tactile P300 BCI on a scale from 0 to 10 (0 = not tiring, 10 = very tiring)?
3. How long do you think you would be able to use the eye tracker/visual/auditory/tactile P300 BCI before needing a break (in hours/minutes)?
4. How satisfied with your own performance are you with the eye-tracker/visual/auditory/tactile P300 BCI on a scale from 0 to 10 (0 = not satisfied at all, 10 = very satisfied)?
5. How satisfied with the system are you (eye-tracker/visual/auditory/tactile P300 BCI) on a scale from 0 to 10 (0 = not satisfied at all, 10 = very satisfied)?

### 2.4. Data acquisition

Electroencephalogram (EEG) data were recorded with a g.Tec g.USBamp (g.Tec GmbH, Austria) with a 0.1–30 Hz bandpass and 50 Hz notch filter with a sampling rate of 256 Hz. Twelve active electrodes (g.Ladybird) were positioned in an electrode cap (g.Gamma) at AF7, FPz, AF8, F3, Fz, F4, C3, Cz, C4, P3, Pz, and P4. The remaining set of four channels could not be used for EEG recordings because the tactile stimulator sent trigger signals to channel 16 and required an independent reference. Data recording, stimulus presentation and signal processing for all four tasks (eye-tracker, visual, auditory and tactile P300 BCI) were implemented using the BCI2000 software package (Schalk et al., 2007) on a Hewlett-Packard EliteBook 840 (HP Inc., USA) with a dual-core CPU (2.5 GHz), 8 GB RAM and a 64-bit Windows 7.

### 2.5. Data analysis

Online classification of the auditory and the visual P300 BCI data were performed using SWLDA (Krusienski et al., 2008). The classifier was trained with a *p*-value threshold of *p* < 0.1 for adding features (forward step) and *p* > 0.15 for removing features (backward step). A maximum of 60 features was selected. The 1000 ms window used for online classification was smoothed with a 20 sample moving average filter and subsampled to every 20th sample. The classifier was applied to single trials and the outputs summed. The symbol with the highest score was selected after 10 repeitions.

Additional offline classification was performed for the auditory, tactile and visual P300 BCI data using shrinkage linear discriminant analysis (SLDA) as suggested in Blankertz et al. (2011). We performed a leave-one-run-out crossvalidation with *n*−1 runs as training data and one run as test data. During each training step gamma coefficients were determined empirically on the training data by using the value that yielded the best accuracy in the range from 0.01 to 0.1 (in 0.01 increments) and from 0.2 to 0.5 (in 0.1 increments). Offline accuracy was calculated for one to 10 stimulus repetitions and 100 to 2,000 ms windows (in 100 ms steps).

For each accuracy the ITR was calculated using the method suggested in Wolpaw et al. (2002). Information transfer per minute was calculated using the selection times described in section 2.2. For each BCI system accuracy was recalculated offline using between one and 10 stimulus repetitions. Based on the ITR the optimal number of stimulus repetitions (highest ITR with an accuracy ≥ 70%) was selected across the average of all participants. Selection times for the eye-tracker were calculated based on the average times needed per selection during the online measurement.

The amplitudes and latencies of the maximal peak of a late positive component in a window from 199 to 1,000 ms (samples 51 to 256) on channels Fz and Cz after stimulus presentation was analyzed for the three BCI systems using EEGLAB (Delorme and Makeig, 2004) and self written scripts under MATLAB. Squared Pearson's correlation coefficients (*r*^2^ values) were used to visualize the data.

### 2.6. Statistical analysis

We performed matched sample *t*-tests to compare visual P300 BCI performance with eye-tracker performance and auditory P300 BCI with tactile P300 BCI performance. Comparisons were restricted to these two pairs because we assume a person with intact vision will choose one of the former and a person without vision one of the latter. A threshold for marginal significance we assume *p* < 0.05, and for high significance *p* < 0.01. For the the aforementioned comparison, we used a Bonferrroni corrected (two comparisons, accuracy and ITR) threshold *p*-value of 0.025 for marginally significant and 0.005 for highly significant results.

The physiological data were compared on a pairwise basis across all three EEG based measures resulting in a total number of twelve comparisons (two electrodes, two measures (amplitude/latency) and three BCIs). We used a Bonferrroni corrected threshold *p*-value of 0.004 for marginally significant and 0.0008 for highly significant results.

As the comparisons of the questionnaire results were again limited to either visual or non-visual systems, we performed pair-wise *t*-tests and used a Bonferrroni corrected (five comparisons, five questions) threshold *p*-value of 0.01 for marginally significant and 0.002 for highly significant results.

## 3. Results

### 3.1. Accuracy and information transfer rate

#### 3.1.1. Visual AACs (visual P300 BCI and eye-tracker)

**Figure 2:**
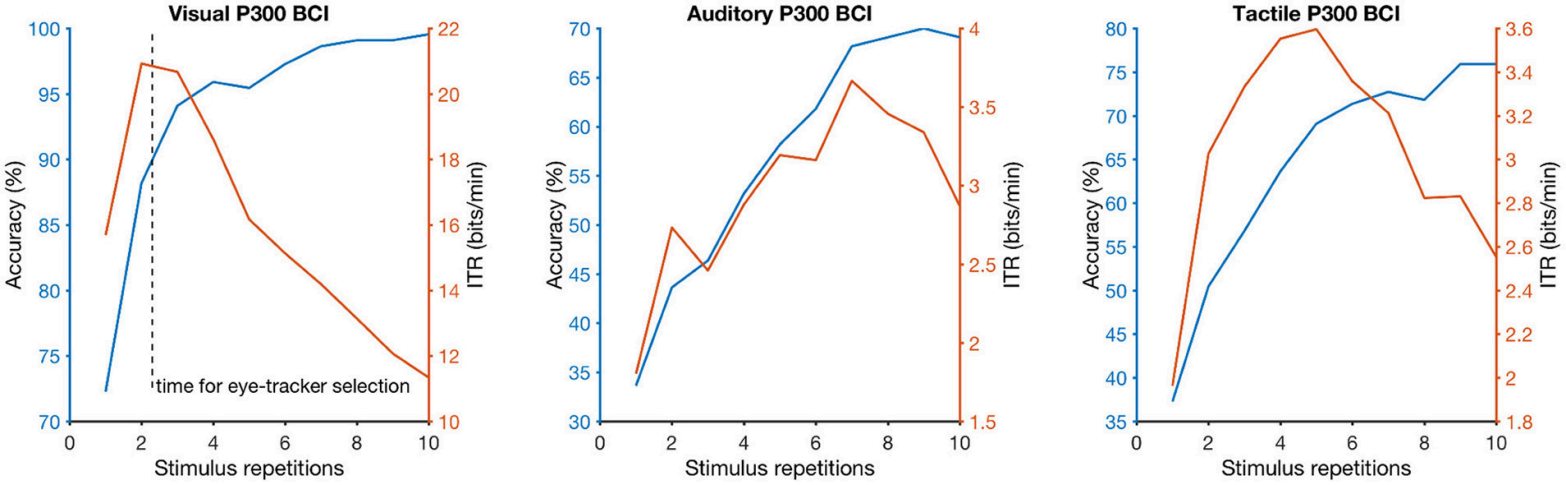
Dependency of selection accuracy (blue line) and information-transfer rate (ITR; orange line) on the number of stimulus repetitions (the more stimulus repetitions the longer a selection needs) for each brain-computer interface (BCI) system (visual on the left, auditory in the center, tactile on the right). The dashed vertical line on the left graph indicates the selection time needed on average when the participants used the eye-tracker (approximately 2 stimulus repetitions or 5 s).

**Figure 3:**
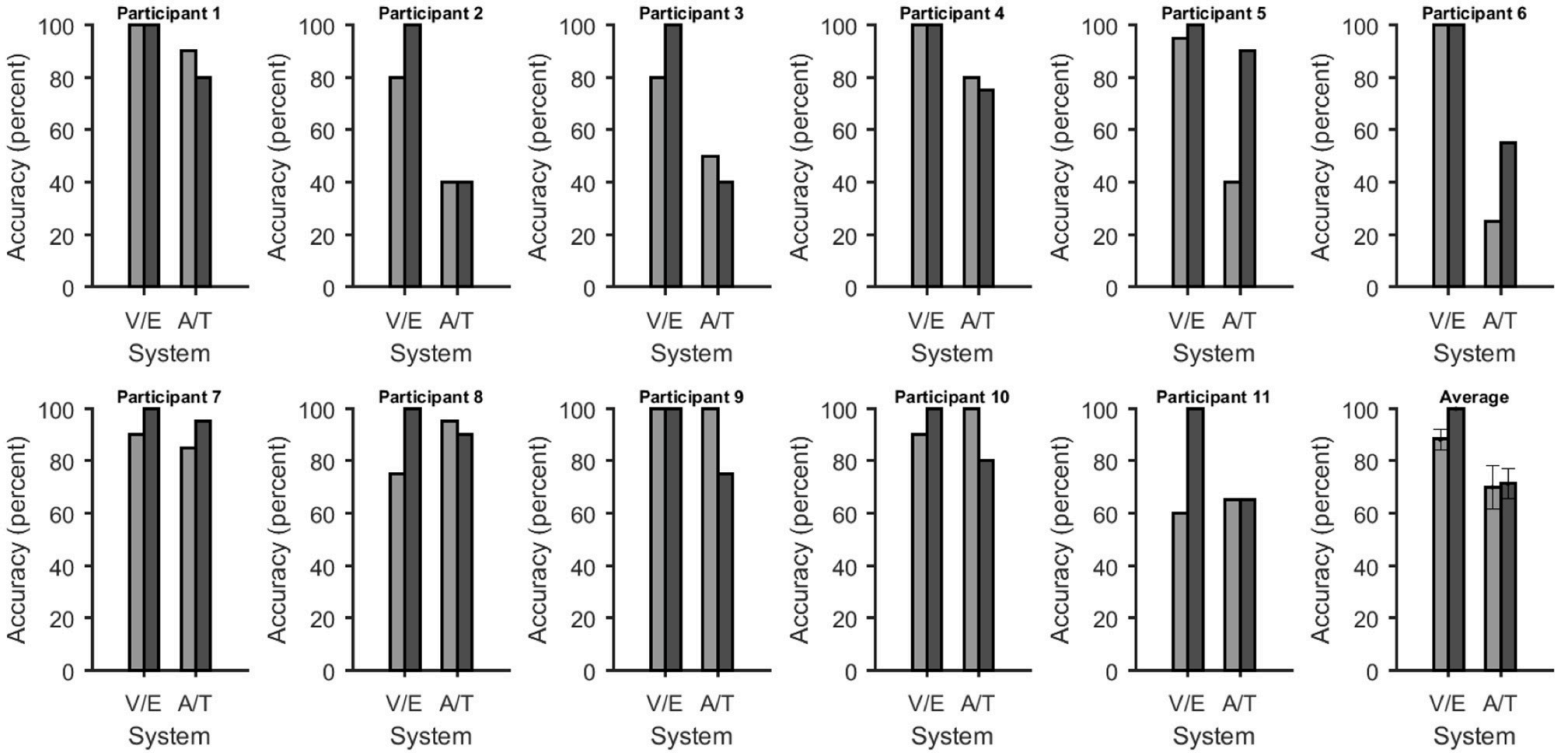
Accuracies at selecting one of five choices for each system and participant. In all figures the bars were grouped according to whether they depend on vision or not [visual P300 BCI (V) with eye-tracker (E) and auditory (A) with tactile (T) P300 BCI]. The average across all participants is shown on the bottom right. Error bars over the plot of the average show the standard error. The accuracy of the eye-tracker system had no variance, thus a *t*-test could no reliably be computed. The accuracies of the non-visual BCIs was not significantly different.

**Figure 4:**
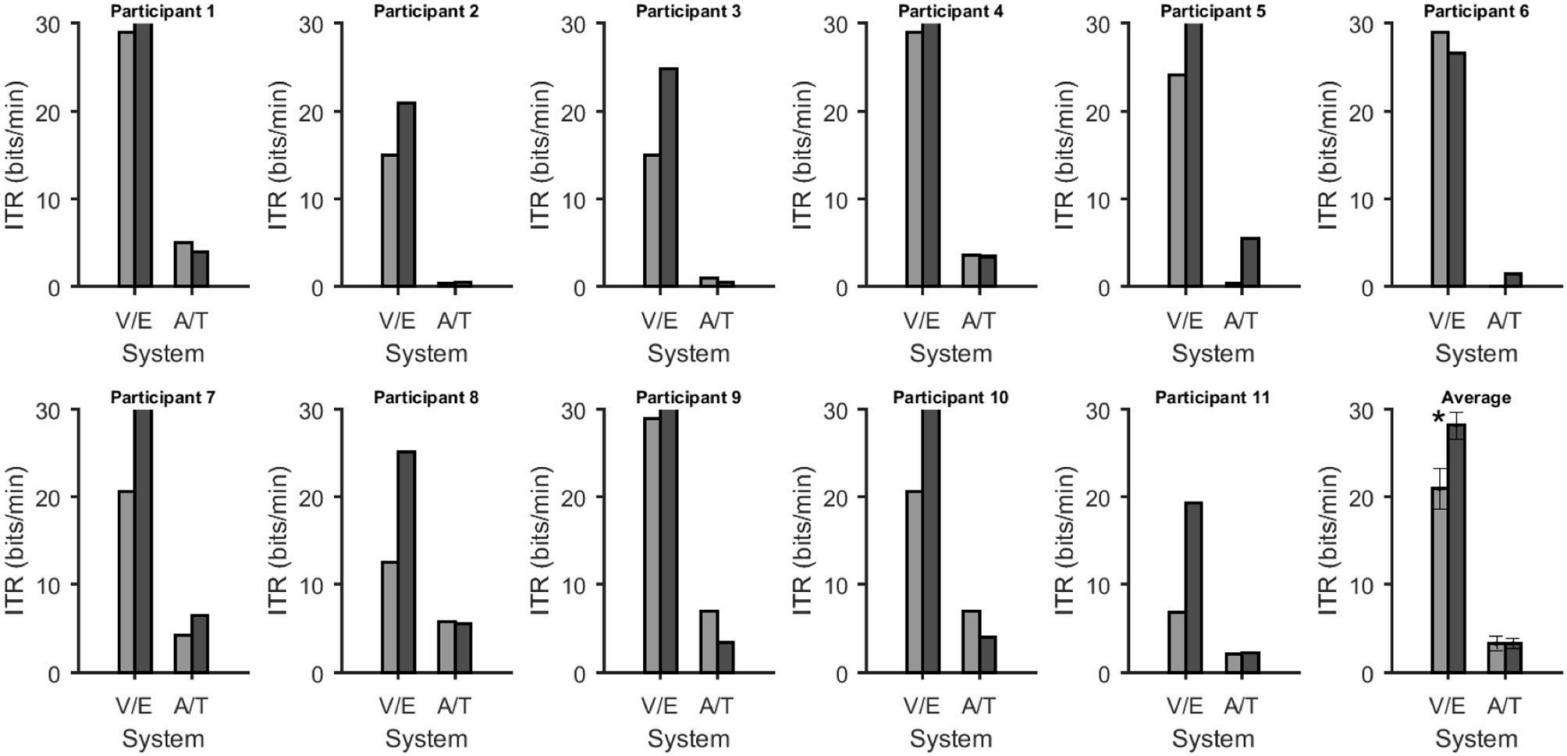
Information transfer rates in bits/min for each system and participant. In all figures the bars were grouped according to whether they depend on vision or not [visual P300 BCI (V) with eye-tracker (E) and auditory (A) with tactile (T) P300 BCI]. The average across all participants is shown on the bottom right. Error bars over the plot of the average show the standard error. The star indicates a highly significant (*p* < 0.005) difference according to a paired *t*-test for matched samples corrected for multiple comparisons.

**Figure 5:**
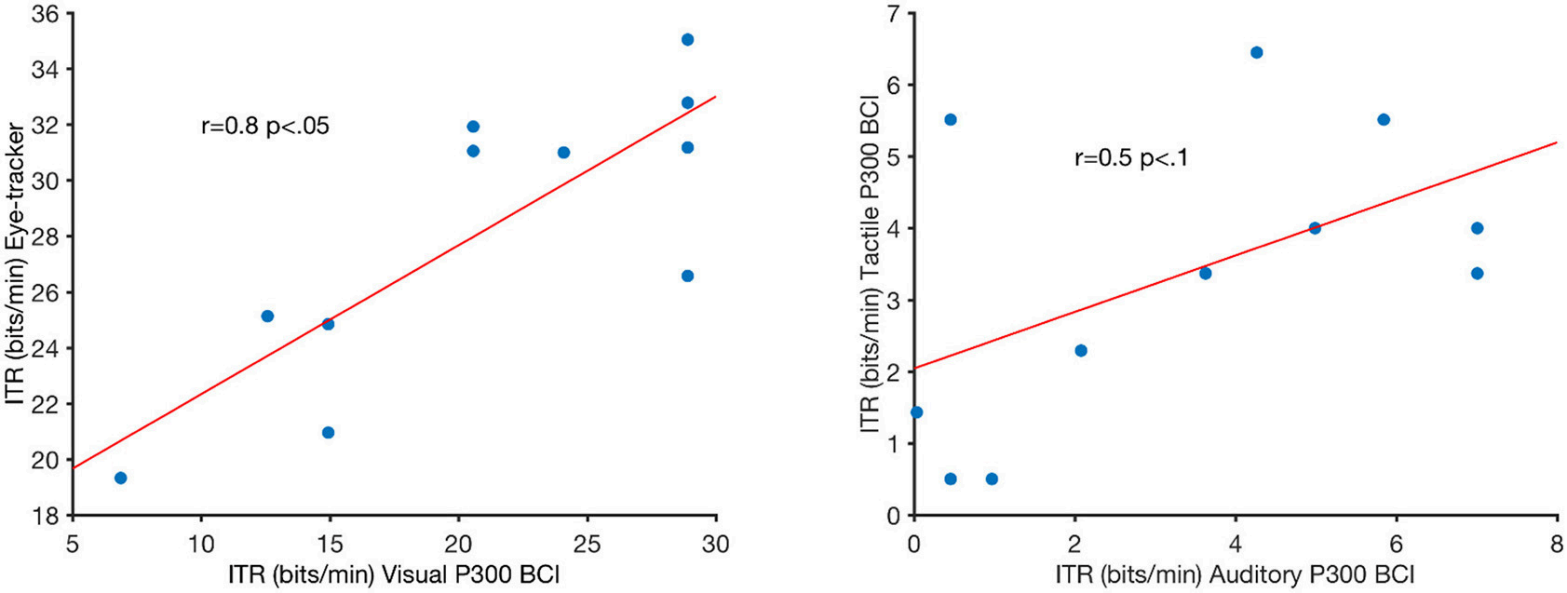
Pearson's correlations of the visual P300 brain-computer interface (BCI) information-transfer rate (ITR) and eye-tracker ITR (left) and auditory P300 BCI ITR and tactile P300 BCI ITR. *R* and *p*-values are indicated in the figure. The red lines are least-square fits on the data.

Online accuracy for the visual P300 BCI was 96% (one participant had less than 100%) and for the eye-tracker 100%. Using the visual P300 BCI with the optimal selection time of two repetitions (4.8 s), determined as shown in Figure 2, the participants selected the targets with an accuracy of 88% (SD 13, range 60–100). This procedure cannot be applied to the eye-tracker and the accuracy was 100% online. Thus, the average time per selection was used to calculate the ITR of the eye-tracker. Interestingly, this time (5.1 s) was only 6% higher than the time needed for two stimulus repetitions (4.8 s) with the visual P300 BCI. Since the variance of the selection times of the BCI systems was very low (e.g., seven participants have the same selection time for the visual P300 BCI since the selection time changes in the discrete steps of time per stimulus repetition) *t*-tests were not performed. The accuracy of the eye-tracking system was 100% for all participants. Since this implies zero variance a *t*-test between the eye-tracking accuracy and the visual P300 BCI accuracy was not calculated. Using the same selection times and accuracies, the ITR of the visual P300 BCI was 20.9 bits/min (SD 7.8, range 6.9–28.9) and for the eye-tracker 28.2 bits/min (SD 5.1, range 19.3–35.1). A *t*-test showed a significant difference between visual P300 BCI and eye-tracker ITR [*t*_(10)_ = −5.1, *p* < 0.005]. The accuracy and ITR results are also shown in Figures 3, 4. Correlation between eye-tracker and visual P300 BCI ITR was high (*r* = 0.8, *p* < 0.05). The scatter plot for this comparison is shown in Figure 5 (left).

#### 3.1.2. Non-visual AACs (auditory and tactile P300 BCI)

Online accuracy for the auditory P300 BCI 58% and for the tactile P300 BCI online performance was not evaluated for technical reasons. Optimal classification rates with regard to ITR were 70% (SD 27, range 25–100) with nine repetitions (19.9 s) for the auditory P300 BCI and of 71% (SD 19, range 40–95) with six repetitions (18 s) for the tactile P300 BCI. Thus, the selection time needed for the auditory P300 BCI was 10% higher than for the tactile P300 BCI. A *t*-test between auditory and tactile P300 BCI accuracy showed no significant differences [*t*_(10)_ = −0.2, *p* = 0.8]. Using the same selection times and accuracies, the ITR of the auditory P300 BCI was 3.3 bits/min (SD 2.7, range 0–7) and of the tactile P300 BCI 3.4 bits/min (SD 2, range 0.5–6.5). A *t*-test showed no difference between auditory and tactile P300 BCI ITR [*t*_(10)_ = 0.0, *p* = 0.98]. The accuracy and ITR results are shown in Figures 3, 4. Correlation between between tactile and auditory P300 BCI ITR was moderate (*r* = 0.5, *p* < 0.1). The scatter plot for this comparison is shown in Figure 5 (right).

### 3.2. Questionnaires

**Figure 6:**
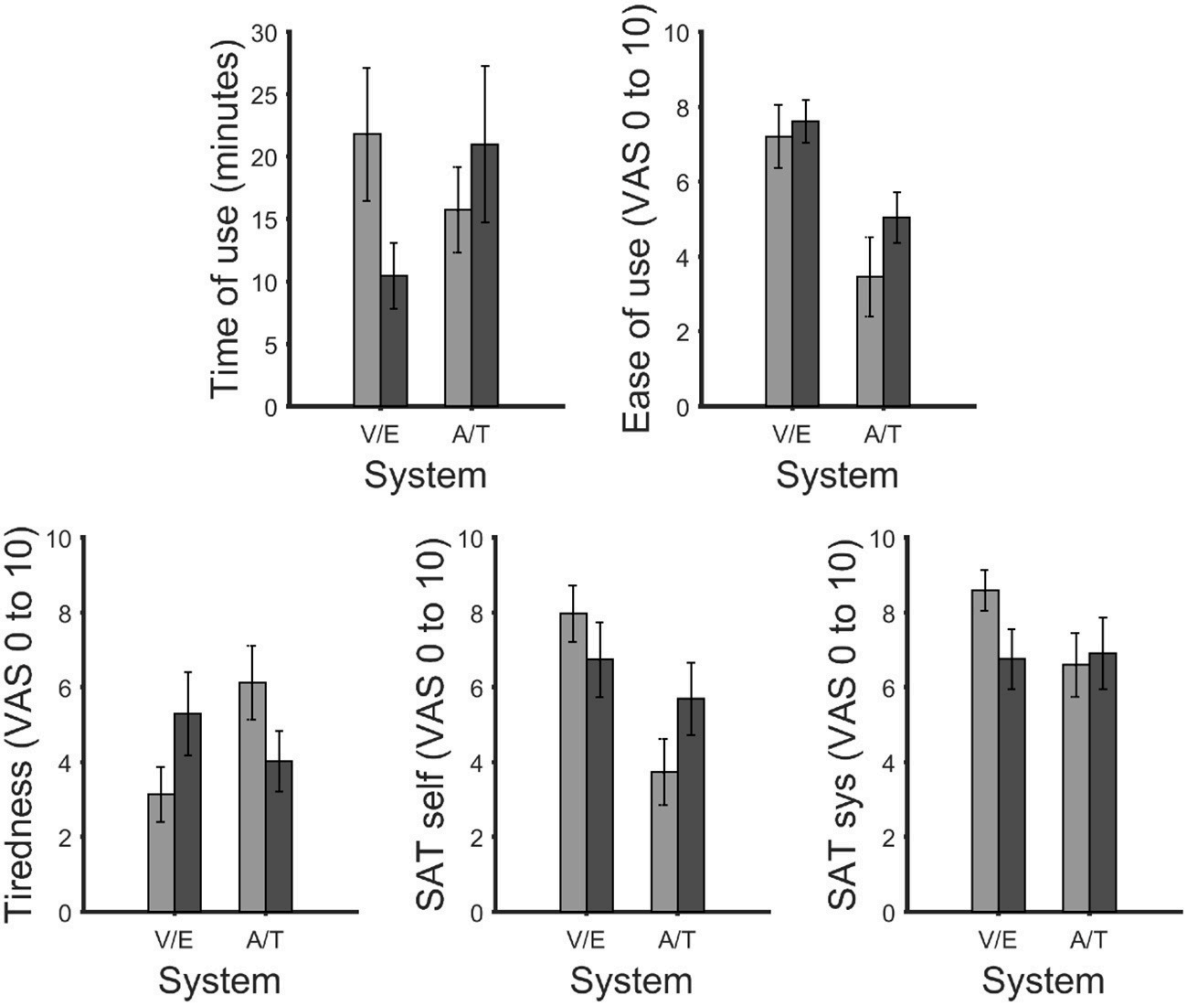
Maximum time of use the participants felt they could use the system without experiencing fatigue (**top left**). Visual analog scale (VAS) ratings for ease of use (**top right**), tiredness (**bottom left**), satisfaction with own performance (**bottom center**) and performance of the system (**bottom right**). After correction for multiple comparisons, no significant differences were found. In all figures the bars were grouped according to vision dependence [visual P300 BCI (V) with eye-tracker (E) and auditory (A) with tactile P300 BCI (T)]. Error bars show the standard error.

The subjective ratings given by the participants included estimated time of use (in minutes; Figure 6), ease of use, tiredness, satisfaction with own performance and satisfaction with the system (all rated on a visual-analogue scale (VAS) from 0 to 10; in Figure 6). After multiple comparison correction none of the differences were significant.

#### 3.2.1. Visual AACs (visual P300 BCI and eye-tracker)

Time of use in minutes between the visual P300 BCI (mean 21.8, SD 17.6, range 2–60) and eye-tracker (mean 10.5, SD 11.3, range 1–40): *t*_(10)_ = 1.9, *p* = 0.08. Ease of use between visual P300 BCI (mean 7.2, SD 2.8, range 1.8–10) and eye-tracker (mean 7.6, SD 1.9, range 3.9–10): *t*_(10)_ = 0.5, *p* = 0.6. Tiredness after using the system between visual P300 BCI (mean 3.2, SD 2.5, range 0–6) and eye-tracker (mean 5.3, SD 3.7, range 0–10): *t*_(10)_ = −2.1, *p* = 0.07. The satisfaction with own performance rating between visual P300 BCI (mean 8.0, SD 2.5, range 1.3–10) and eye tracker (mean 6.7, SD 3.3, range 0.2–10): *t*_(10)_ = 2.2, *p* = 0.05. Finally, the satisfaction with the system between visual P300 BCI (mean 8.6, SD 1.8, range 4.1–10) and the eye-tracker (mean 6.8, SD 2.7, range 1.6–10): *t*_(10)_ = 2.8, *p* = 0.02.

#### 3.2.2. Non-visual AACs (auditory and tactile P300 BCI)

Time of use in minutes between the auditory P300 BCI (mean 15.7, SD 8.7, range 2–30) and tactile P300 BCI (mean 21, SD 20.8, range 3–60): *t*_(10)_ = −2.1, *p* = 0.07. Ease of use between auditory P300 BCI (mean 3.5, SD 3.5, range 0–8.8) and tactile P300 BCI (mean 5.1, SD 2.3, range 1.2–9): *t*_(10)_ = −1.9, *p* = 0.09. Tiredness after using the system between the auditory P300 BCI (mean 6.1, SD 3.3, range 0–9.2) and tactile P300 BCI (mean 4.0, SD 2.7, range 0–7.4): *t*_(10)_ = 2.6, *p* = 0.03. The satisfaction with own performance rating between the auditory P300 BCI (mean 3.7, SD 2.9, range 0.2–9.6) and the tactile P300 BCI (mean 5.7, SD 3.2, range 0.8–10): *t*_(10)_ = 2.2, *p* = 0.06. Finally, the satisfaction with the system between auditory P300 BCI (mean 6.6, SD 2.8, range 1–10) and tactile P300 BCI (mean 6.9, SD 3.2, range 1–10): *t*_(10)_ = −0.6, *p* = 0.6.

### 3.3. Physiological data

**Figure 7:**
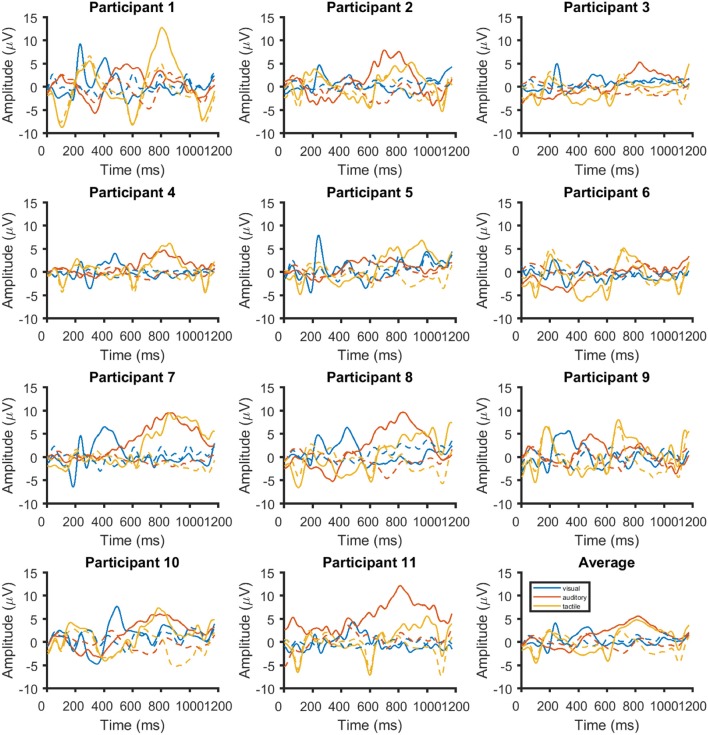
Time course of the event-related potentials on Cz for each of the 11 participants and the average across all (bottom right). Responses are shown in blue for visual stimuli, red for auditory stimuli and yellow for tactile stimuli. The responses for ignored stimuli are shown with dashed lines.

**Figure 8:**
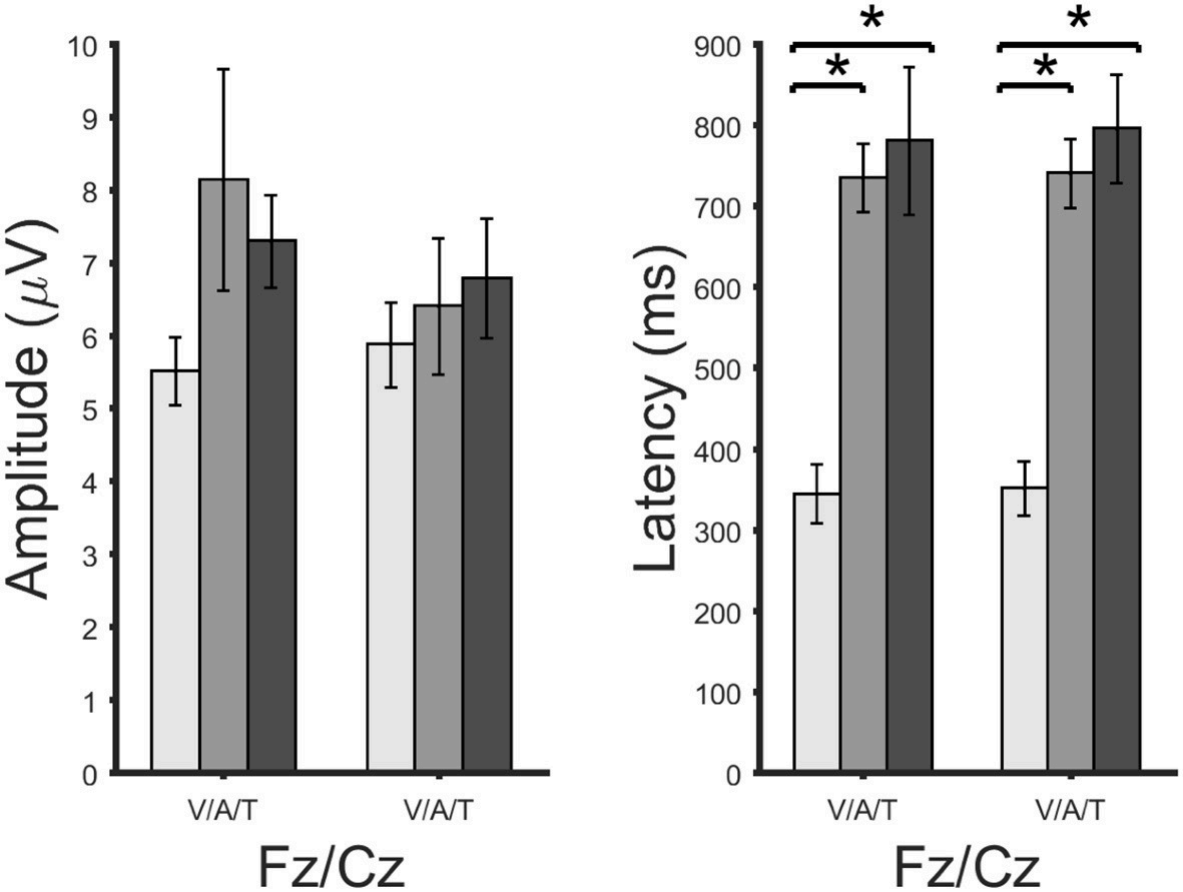
Amplitudes (**right**) and latencies (**left**) of a late positive component shown for each brain-computer interface system and channels Fz and Cz. Significant differences according to a paired *t*-test for matched samples are indicated by the black lines. A large star highly significant difference (corrected *p*-values of 0.0008). Error bars show the standard error.

Figure 7 the ERPs from channel Cz for all systems and participants individually and as an average. The visual ERPs have a lower latency than both the auditory and tactile ERPs. Topographically, the auditory ERPs have a more frontal orientation than the tactile ERPs with the amplitudes and latencies being similar. Amplitude and latency values of a late positive component are listed in detail in Table 1. As shown in Figure 8 only the latency differences between the visual and the non-visual ERPs [visual vs. tactile on Fz: *t*_(10)_ = −5.3, *p* < 0.0008; visual vs. auditory on Fz: *t*_(10)_ = −10.2, *p* < 0.0008; visual vs. tactile on Cz: *t*_(10)_ = −7.1, *p* < 0.0008; visual vs. auditory on Cz: *t*_(10)_ = −9.5, *p* < 0.0008] were highly significant.

**Table 1:** Amplitude and latency values of the maximal peak of a late positive component between in a window from 199 to 1,000 ms (samples 51 to 256 sampled at 256 Hz).

	**Visual P300 BCI**			**Auditory P300 BCI**			**Tactile P300 BCI**		
	**Mean**	**SD**	**Range**	**Mean**	**SD**	**Range**	**Mean**	**SD**	**Range**
Fz (amplitude)	5.5 μV	1.6	3.7–7.7	8.1 μV	5	2.4–15.2	7.3 μV	2.1	3.8–10.7
Fz (latency)	341 ms	120	219–488	732 ms	141	477–887	778 ms	303	199–1,000
Cz (amplitude)	5.9 μV	1.9	2.8–9.3	6.4 μV	3.1	1.8–12.2	6.8 μV	2.7	2.2–12.8
Cz (latency)	348 ms	113	227–492	737 ms	140	484–871	792 ms	222	199–996

### 3.4. Influence of classification time window

**Figure 9:**
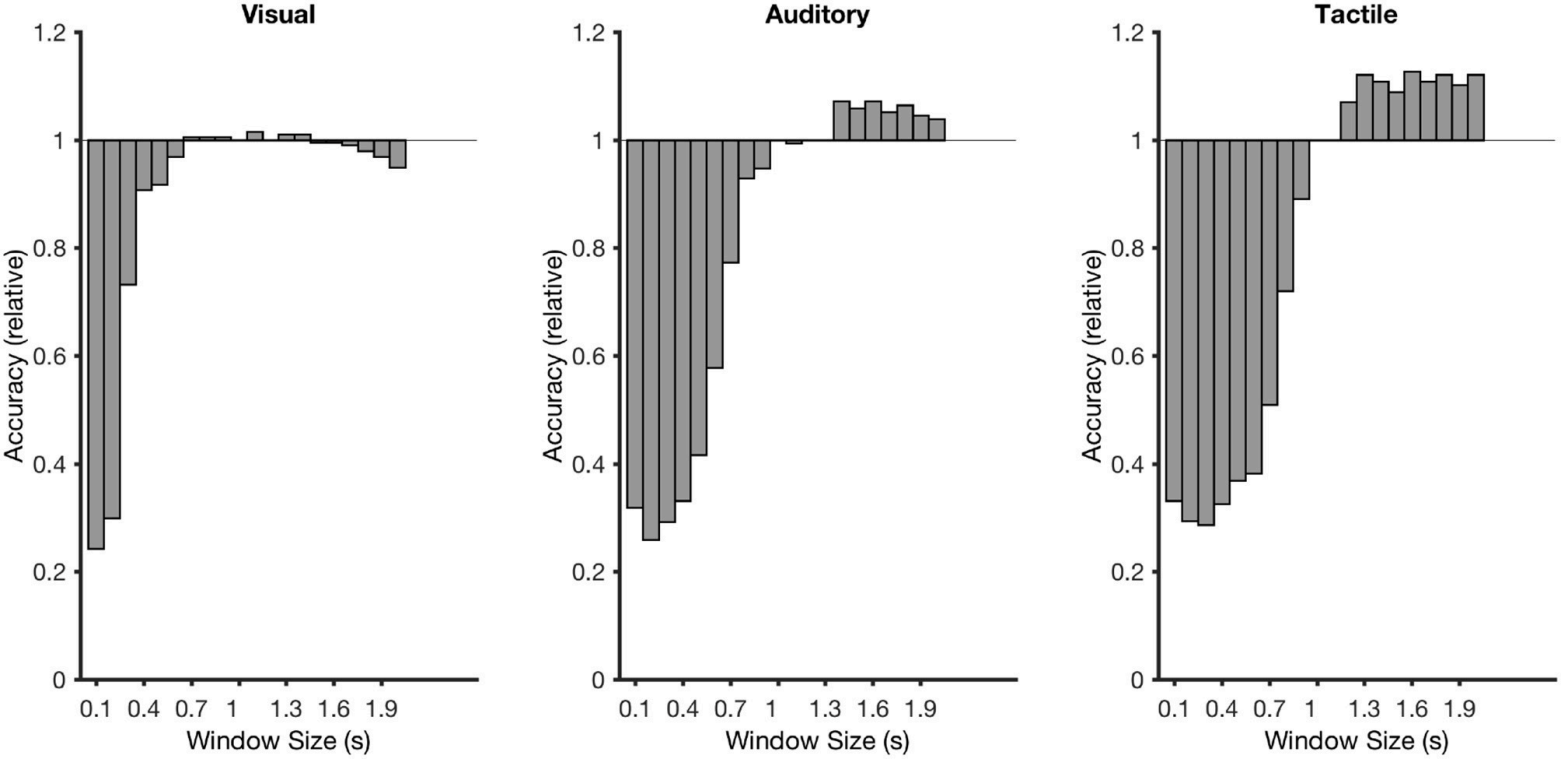
Recalculated classification rates for time windows between 100 and 2,000 ms. The classification rates were calculated as a factor in relation to the accuracy with a window of 1,000 ms.

Figures 7, 8 showed that there was a considerable differences in latencies between visual and non-visual P300 peak latencies. Performing the cross-validation that was applied in section 3.1 with ERP window lengths between 100 and 2,000 ms and using the accuracy obtained at 1,000 ms as a reference showed that longer windows lead to an average improvement of classification performance of 13% with a window of 1600 ms for the tactile P300 BCI and of 7% with a window of 1,400 ms for the auditory P300 BCI. With smaller classification windows the accuracies decrease for all three BCIs (see Figure 9). A *t*-test showed a moderate difference between the 1,000 and 1,400 ms for the auditory P300 BCI [*t*_(10)_ = −2, *p* < 0.1] and a difference between the 1,000 ms and 1,600 ms window for the tactile P300 BCI [*t*_(10)_ = −3.8, *p* < 0.05]. The ITRs (calculated for each participant individually and then averaged) increased to 4.3 bits/min (ITR increased by 30%) at 7 repetitions and an accuracy of 73% for the auditory P300 BCI and to 4.6 bits/min (ITR increased by 35%) at 4 repetitions and an accuracy of 72% for the tactile P300 BCI.

## 4. Discussion

We investigated four different AAC systems: visual, auditory and tactile P300 BCIs and an eye-tracker. We compared the performance on the basis of accuracy and ITR, differences in the ERPs on the basis of amplitude and latency and finally the subjective initial impression of the users on the basis of a short questionnaire.

### 4.1. Performance

Using an adapted input system with word completion experienced users can write up to 25 words per minute using an eye-tracker (Ward and MacKay, 2002). Inexperienced users in the first 10 minutes of usage wrote between 5 and 10 words per minute. Considering the system used word completion it is not certain how many selections were needed to write five words but it is a minimum of five. In our study users (which were also inexperienced and in the first 10 minutes of using an eye-tracker) needed 5 s to make one selection with the eye-tracker enabling them to make 12 selections per minute. Even though it is not possible to make an exact comparison we consider this to be of a similar order of magnitude and thus representative of what can be achieved using an eye-tracking system. Nonetheless, there are possibilities to optimize the parameters used to determine gaze and thus selection accuracy and speed which we did not do (Mack et al., 2017).

Online the visual P300 BCI was the only system, besides the eye-tracker, with which most of the users (10 of 11) selected 100% of the symbols correctly. Offline 7 out of 11 participants selected over 90% of the symbols correctly. The ITR was 20.9 bits/min (eye-tracker 28.2 bits/min). Compared to highly optimized visual BCI systems this is not the maximum: over 100 bits/min were demonstrated in Spüler et al. (2012), Kaufmann and Kübler (2014), and Townsend and Platsko (2016). Nonetheless, the use of face stimuli and a regularized linear discriminant analysis (LDA) employs are optimizations to the visual P300 BCI paradigm (Blankertz et al., 2011; Kaufmann et al., 2013b). Optimizing the selection time online, which we performed offline, is performed by the eye-tracking system (no selections were made before the criteria were met) and could be integrated into the P300 BCI (Schreuder et al., 2013). Additionally, the visual P300 BCI system would benefit from an asynchronous mode of operation as is integrated into the eye-tracking system (Pinegger et al., 2015).

In our study the ITR of the non-visual BCIs was lower (auditory: 3.3 bits/min; tactile 3.4 bits/min) than with the visual systems. This was an expected result that was also shown in many previous studies (for a review see Riccio et al., 2012). It was shown that training can increase the performance of both auditory and tactile systems (Baykara et al., 2016; Herweg et al., 2016), whereas no effect of session was found in visual P300 BCI studies (Nijboer et al., 2008). Additionally, in out study the ITR of the non-visual systems was increased by 30% by choosing a longer classification window length. An effect of classification window length was also described in Kaufmann et al. (2013a), showing a dependency of increasing or decreasing accuracy on stimulus and inter-stimulus-interval duration. Nonetheless, even if a person uses either the auditory or the tactile system for a longer period of time the ITR will not increase to the level of the visual systems. There are two main reasons for this difference in speed. One, presenting an auditory or tactile stimulus takes a longer amount of time (stimulus onset asynchrony of the visual P300 BCI was 187.5 ms compared to 375 ms of the auditory and 500 ms of the tactile P300 BCI). And two, ignoring the non-target stimuli is harder with non-visual systems because each stimulus has to be attended to determine if it is the target stimulus. In the visual case, non-targets can be attenuated using gaze. Additionally, fixating a visual stimulus with gaze increases the VEPs this stimulus elicits in comparison to non-fixated stimuli. This is an additional feature that can be used by classifier. Thus, if the user fixates a central position on the screen, and not individual stimuli, performance decreases (Brunner et al., 2010).

A surprising result was that after determining the selection time that yields the highest ITR and an accuracy above 70% for the visual P300 BCI we found that the time needed for an average selection with the eye-tracker was only 6% higher (4.8 s with the BCI compared to 5.1 s with the eye-tracker). Furthermore, the ITR was highly correlated (*r* = 0.8). Thus, our results indicate that both the visual BCI and the eye-tracker require visual search and thus the aptitude users exhibited for these tasks was correlated. Essentially both tasks could be called top-down volitional attentional selection processes (Itti and Koch, 2000). Using optogenetic activation in mice, Zhang et al. (2014) showed that visual selective attention task performance increases with the activity in the cingulate cortex. On a behavioral level, Najemnik and Geisler (2005) showed that visual search depends on an efficient strategy to determine fixation locations. It is possible that performance of both the visual BCI and the eye-tracker task was governed by the users ability to maintaining visual attention and using an optimal search strategy for the fixation locations (the targets). Since the performance between auditory and tactile BCI was not strongly correlated it may be beneficial to combine tactile and auditory stimulation in one BCI paradigm (Rutkowski and Mori, 2015). A recent study also showed performance gains when combining eye-tracker with visual P300 BCI compared to either visual P300 BCI or eye-tracker only (Kalika et al., 2017).

Pasqualotto et al. (2015) showed approximately 48% increase in ITR from visual P300 BCI to eye-tracker whereas we found an increase of of 34%. Considering the task and sample difference we think this is fairly similar. A remarkable difference between the two studies is how the participants rated the use of visual P300 BCI and eye-tracker subjectively. In Pasqualotto et al. (2015) rated the usability of the eye-tracker as higher and the workload lower whereas in the current study users were more satisfied with the P300 BCI and also felt it was less tiring. There may be two possible reasons for this. One, the eye-tracking task was always performed last in our study which may have led to an increase of tiredness. The sequence of task does not universally determine this though, because the auditory BCI was performed before the tactile BCI and rated as more tiring. The second reason may be the optimized stopping method used by the eye-tracker, i.e., the users were asked to move to the next symbol as quickly as possible after hearing the affirmative sound. In case of the visual P300 BCI the users attended the symbols for a fixed amount of time. Consequently, using an optimized stopping method may be too demanding for first time users. A dependency of the performance difference between visual BCIs and eye-tracker performance on target size was shown by Suefusa and Tanaka (2017). The authors showed that in an SSVEP BCI paradigm the BCI's ITR surpassed that of an eye-tracker for smaller target sizes and suggest that the interface should be chosen depending on the size of the targets that can be selected by the user.

From a practical point of view the BCI systems need the EEG cap, whereas the eye-tracker does not depend on a lengthy setup time. Nonetheless, the eye-tracker may need to be recalibrated frequently due to changing conditions. Regular recalibration of the BCI system may be an advantage during the training phase (Baykara et al., 2016).

### 4.2. Physiological data

Latencies between the visual (about 300 ms) and the non-visual BCIs (about 700 ms) were different for both Fz and Cz. This is not an unexpected result and was shown before in BCI studies (Furdea et al., 2009; Klobassa et al., 2009; Halder et al., 2013) as well as non-BCI studies (Kotchoubey and Lang, 2003). As mentioned before, training not only increases ITR but also decreases latency of the auditory P300 (Baykara et al., 2016). Only an increase in amplitude but not a decrease in latency was reported in Herweg et al. (2016) for tactile P300 BCIs, but since the latency was not discussed it is currently unclear whether P300 peak latency will decrease with training in a tactile P300 BCI task. In Baykara et al. (2016) the latency decreased by about 50 ms, and in Halder et al. (2016b) by about 60 ms. In a study with end-users a decrease in latency was found for one out of five participants whereas three out of five showed increases in amplitude of the late positive component (Halder et al., 2016a). Compared to other studies using auditory stimulation, the latencies we found were high. For example in Baykara et al. (2016) the latencies were at about 500 ms where we measured latencies of the peak amplitude at around 700 ms. This may be due to the stimulus material as Baykara et al. (2016) used the sounds from Simon et al. (2014) where the peak latencies were measured at 350 ms. In our previous study using the same Hiragana syllables as in this study, latencies were also around 700 ms (Halder et al., 2016b). Thus, in case of the auditory P300 BCI the high latencies were not unexpected. Visual inspection of the tactile ERPs in Herweg et al. (2016) shows latencies at about 500 ms, which is also what was reported in Kaufmann et al. (2014). In the aforementioned study, the authors used an on-time of 220 ms and an off-time of 400 ms. We used an on-time and off-time of 250 ms. Assuming a peak with a latency of 500 ms in our sample, the onset of the next stimulus after a target stimulus after 500 ms may attenuate this peak because the negative early negative potentials elicited by the tactile stimulation were quite strong (around −4 μV, see Figure 7). The conclusion may be that either stimulus onset asynchronies below 500 ms (as suggested in Brouwer and van Erp, 2010) or above 500 ms (as used in Kaufmann et al., 2014) may lead to better results than the 500 ms used in the current study.

### 4.3. Questionnaire

Our sample size of 10 participants suggests that the results of the questionnaire should be interpreted with caution. Our users felt the ease of use of the two visual systems to be equal wheres the tactile BCI was rated to be slightly easier to use than the auditory BCI. Furthermore, the auditory BCI was found to be more tiring than the tactile BCI. Interestingly, the users felt the eye-tracker is more tiring than the visual BCI. This may be due to the fact that the eye-tracker was always performed last but also to the fact that the users felt pressured by the asynchronous design. The design forced the user to perform the task as fast as they could and not at the speed of the system which put more pressure on the users. This is supported by the third questionnaire in which the users were asked how satisfied with their own performance. Most users reported to be more satisfied with their own performance with the visual BCI than with the eye-tracker. This may be due to the fact that the users felt they could have been faster with the eye-tracker. Also, the users were more satisfied with their own performance with the tactile BCI than with the auditory BCI. Concerning the satisfaction with the system, again the users were more satisfied with the visual BCI than with the eye-tracker. Auditory and tactile were rated to be equal. In summary, these data indicated a slight preference of the users for the visual BCI and the tactile BCI compared to their respective counterparts. The results may be influenced by the eye-tracker always having been performed last and the eye-tracker being asynchronous (which we initially thought to be an advantage for the eye-tracker). Concerning the usability of the tactile system, one user stated after the measurement that it was uncomfortable that you could not move your fingers for an extended period of time.

As mentioned before these results should be interpreted with caution as none of the statistical tests were significant after multiple comparison corrections. As a consistent trend though the users seem to be less satisfied with the eye-tracker than with the visual P300 BCI. This may be because the asynchronous behavior of the eye-tracker was not perceived as an advantage by the current sample of participants. This indicates that such a feature should be introduced only after the users have familiarized themselves with the system (Lotte et al., 2013). As a consequence, the initial impression of the users we presented the visual systems to was that the visual BCI could be used for a longer time and be less tiring. Additionally the users felt that their own performance and the BCI system itself was better. Again this may be due to the difference in behavior and also sequence effects. Even though performance of both non-visual systems was identical, the users found the tactile BCI to be usable longer and easier, less tiring and thought their own performance was better.

## 5. Conclusions

In summary, our participants achieved the highest accuracy and ITR with the eye-tracker, followed by the visual P300 BCI. All participants were able to select 100% of the choices correctly using the eye-tracker, 10 out of 11 with the visual P300 BCI. As could be expected based on previous work performance of the auditory and tactile BCIs was lower. Performance of eye-tracker and visual P300 BCI was highly correlated whereas performance of the auditory and tactile BCI was only slightly correlated. This leads us to conclude that choosing the most suitable non-visual BCI has a larger impact than choosing the eye-tracker or visual P300 BCI.Whereas most users had slightly higher ITRs with the eye-tracker, the number of users with higher auditory BCI performance was six (correspondently five had higher tactile BCI performance).

We think one of the most astounding results of the current study was the strong correspondence between the two vision dependent systems (the ideal selection time differed only by 5%) and the vision independent systems (ideal selection time differed by 10%) whereas the difference between the two categories was substantial (~400%). This indicates that both systems of each category may be considered for the same tasks and the user preference and individual abilities should be the decisive factor.

Both visual P300 BCI and eye-tracker can provide very high communication speeds in this task with a sample of healthy participants. Tactile BCI and auditory BCI showed on average identical ITR. We will investigate further with end-users with motor impairments in which scenarios the use of eye-trackers and in which the use of BCIs offers the greatest advantage.

## Author contributions

SH planned experiment, collected data, performed analysis and wrote manuscript. KT planned experiment, collected data and wrote manuscript. KK planned experiment and wrote manuscript.

### Conflict of interest statement

The authors declare that the research was conducted in the absence of any commercial or financial relationships that could be construed as a potential conflict of interest.
